# Insights into the Effect of Light Pollution on Mental Health: Focus on Affective Disorders—A Narrative Review

**DOI:** 10.3390/brainsci14080802

**Published:** 2024-08-10

**Authors:** Giulia Menculini, Federica Cirimbilli, Veronica Raspa, Francesca Scopetta, Gianmarco Cinesi, Anastasia Grazia Chieppa, Lorenzo Cuzzucoli, Patrizia Moretti, Pierfrancesco Maria Balducci, Luigi Attademo, Francesco Bernardini, Andreas Erfurth, Gabriele Sachs, Alfonso Tortorella

**Affiliations:** 1 Section of Psychiatry, Department of Medicine and Surgery, University of Perugia, 06132 Perugia, Italy; f.cirimbilli@gmail.com (F.C.); veronica.raspa1@unipg.it (V.R.); francesca.scopetta@yahoo.it (F.S.); gianmarco.cinesi@gmail.com (G.C.); anastasia.chieppa@gmail.com (A.G.C.); lorenzocz@libero.it (L.C.); patrizia.moretti@unipg.it (P.M.); balducci.pierfrancesco@gmail.com (P.M.B.); alfonso.tortorella@unipg.it (A.T.); 2 CSM Terni, Department of Mental Health, Local Health Unit USL Umbria 2, 05100 Terni, Italy; 3 Department of Mental Health, North West Tuscany Local Health Authority, 57023 Cecina, Italy; luigi.attademo@hotmail.it; 4 SPDC Pordenone, Department of Mental Health, AsFO Friuli Occidentale, 33170 Pordenone, Italy; francescobernardini78@yahoo.fr; 5 Department of Psychiatry and Psychotherapy, Medical University of Vienna, 1090 Vienna, Austria; andreas.erfurth@meduniwien.ac.at (A.E.); gabriele.sachs@meduniwien.ac.at (G.S.); 6 Klinik Hietzing, 1st Department of Psychiatry and Psychotherapeutic Medicine, 1130 Vienna, Austria

**Keywords:** light pollution, artificial light at night, urbanization, mental health, circadian rhythms, mood disorders, psychopathology

## Abstract

The presence of artificial light at night has emerged as an anthropogenic stressor in recent years. Various sources of light pollution have been shown to affect circadian physiology with serious consequences for metabolic pathways, possibly disrupting pineal melatonin production with multiple adverse health effects. The suppression of melatonin at night may also affect human mental health and contribute to the development or exacerbation of psychiatric disorders in vulnerable individuals. Due to the high burden of circadian disruption in affective disorders, it has been hypothesized that light pollution impacts mental health, mainly affecting mood regulation. Hence, the aim of this review was to critically summarize the evidence on the effects of light pollution on mood symptoms, with a particular focus on the role of circadian rhythms in mediating this relationship. We conducted a narrative review of the literature in the PubMed, Scopus, and Web of Science datasets. After the screening process, eighteen papers were eligible for inclusion. The results clearly indicate a link between light pollution and the development of affective symptoms, with a central role of sleep disturbances in the emergence of mood alterations. Risk perception also represents a crucial topic, possibly modulating the development of affective symptoms in response to light pollution. The results of this review should encourage a multidisciplinary approach to the design of healthier environments, including lighting conditions among the key determinants of human mental health.

## 1. Introduction

For the first time in human history, the majority of the population worldwide lives in urban areas. Indeed, more than 50% of people throughout the world live in cities, and this is expected to increase by 2050 (to around 70%), with urban areas reaching 500,000 inhabitants or more [1]. As previously demonstrated, living in an urban environment significantly increases the exposure to several risk factors, such as different sources of pollutants (e.g., air, noise, light pollution), with a subsequent significant impact on human health [2]. The different sources of pollution contribute to the development of psychological distress and a higher risk for psychiatric disorder onset or exacerbation. In particular, affective disorders generally show a higher prevalence in urban centers. An increase of 21% has been reported for anxiety disorders and more than 39% [3,4] for mood disorders. Studies with different experimental designs have also suggested a higher risk of schizophrenia among urban inhabitants [5,6,7]. The physiological mechanisms behind these observations have largely been explored in an attempt to identify putative biological links between the urban environment and mental health. To this purpose, previous research has documented the role of changes in brain function based on neuroimaging techniques [8]. In particular, urban living and social stress appeared to be associated with increased activation of the amygdala and the anterior perigenual cingulate cortex [9]. More recently, research has addressed epigenetic regulation in preclinical and clinical settings, showing the influence of these mechanisms on the development of specific behavioral phenotypes [10,11,12]. The term “light pollution” was first used in the 19th century, although the initial attempts to describe and analyze this phenomenon were made by observing the effects of artificial light at night on bird migration. Indeed, not only does light pollution affect the well-being of humans but it also plays a significant role in exacerbating ecological crises worldwide by causing severe damage to both plant and animal populations [13]. The awareness of the problem of light pollution among scientists has increased considerably and, as a result, the number of available satellite images has increased. About 83% of the world’s population lives in areas that are highly polluted by artificial light [14]. The greatest intensity of this phenomenon can be detected in urban centers, especially those with high population density. Artificial light at night (ALAN) is suspected of having negative effects on humans, animals, plants, and the balance of ecosystems. ALAN can affect the health and well-being of urban residents, as demonstrated by the increasing number of studies on the effects of light pollution on human health [15]. The light–dark cycle plays a significant role in the regulation of human circadian rhythms. Circadian rhythms are endogenous daily variations in physical, mental, and behavioral activity, which can synchronize with geophysical time showing entrainability. The suprachiasmatic nucleus (SCN), located in the anterior hypothalamus, acts as a master pacemaker, ensuring the best adaptation to both external and internal environments by constantly adapting to the feedback it receives [16]. The secretion of melatonin, a hormone associated with the control of circadian rhythms, is regulated by the conditions of external illumination; melatonin is released in the evening and suppressed by exposure to morning light [17]. The production of this hormone by the pineal gland is regulated by information stimulating the retinal photoreceptors, which are sensitive to different wavelengths [18]. There is evidence of a significant suppression of melatonin secretion after two hours of exposure to monochromatic light at 460 nm late in the evening [14,19]. Under the same conditions, with a wavelength of 550 nm, such effects were not observed. A strong inhibitory effect applies to short wavelengths and color temperature of about 6500 K; indeed, the use of light below 3000 K and the reduction of the blue spectral color is recommended [18]. Inappropriate spectral characteristics also exacerbate skyglow. In particular, 4000 K white LED is 2.5 times more polluting than low-temperature lighting, assuming the same photopic flow and upward emission function [14,15,19]. Light pollution is an emerging issue, and in some countries, there is still a lack of legislation regulating emissions. This may lead to poor choices of infrastructure and lighting fixtures that are harmful to the environment and towards human health. Exposure to ALAN is estimated to have increased by 3–6% annually in recent decades and continues to expand further in space, time, and intensity with more than 2% annual growth in radiance and extent [18]. Increased awareness of the risks that light pollution can pose to psychophysical health would also help to implement preventive behaviors, such as individual initiatives to use eye masks or lower blinds in the evening. However, increased environmental awareness can also lead to concerns about the future [20]. This concern about environmental issues affecting human health is referred to as "eco-anxiety," which is the anxiety people experience about environmental conditions [21,22]. The negative effects of eco-anxiety that have been found in different countries have contributed to a deeper understanding of the relationship between environmental issues and individual behavior [22]. As for the specific influence of light pollution on human health, the physiological effects of exposure to ALAN can both be direct and indirect. Direct physiological effects involve the disruption of circadian rhythms and, in particular, the release of melatonin, a hormone that regulates changes in physiological processes, e.g., body mass, metabolic rate, hormone synthesis, and immunity, as a function of day length [19]. Melatonin has also been demonstrated to exert anxiolytic effects in animal experiments and several clinical conditions. Therefore, ensuring sufficient melatonin production by reducing ALAN and maintaining a regular sleep schedule may represent an endogenous anxiolytic system itself [23]. Based on the above, ALAN may contribute to the development or worsening of obesity and metabolic disorders [24], and is associated with an increased risk of developing breast cancer in women, prostate cancer in men, and pancreatic cancer in both [25,26,27,28]. Although the mechanisms are still unclear, changes in the physiological release and suppression of melatonin are likely to be involved in the development of these conditions [14]. ALAN may also have indirect effects on other physiological processes. For example, components of the immune system have circadian clocks and are therefore likely to be vulnerable to the effects of ALAN. The weakening of the immune system caused by external light pollution may make the human body more susceptible to viral infections [29]. Furthermore, it is discussed to what extent ALAN, which can cause sleep deprivation, may also have further indirect effects on cardiovascular and endocrine functions [30]. Indeed, the disruption of the physiological exposure to natural light, e.g., as a consequence of blue light from electronic devices (such as tablets, PCs, smartphones), has been shown to have negative effects on sleep in particular, with a decrease in sleep quality and sleep duration [31]. Despite the above evidence, more research is needed on the effects of light pollution on mental health in terms of affective symptoms and disorders. Moreover, a better understanding of the role that circadian disruption may play in the development of such conditions would be of help to clinicians in order to implement prevention and treatment strategies. 

This review aims to identify possible effects of light pollution on mental health, with a particular interest in affective symptoms and disorders. Indeed, the effects of light pollution on human health are less well documented than those of other pollutants. Our hypothesis is that artificial light may affect mental health by contributing to the development of affective symptoms and disorders through alterations in circadian rhythms.

## 2. Materials and Methods

A literature search in the online databases PubMed, Scopus, and Web of Science was performed up to May 31st, 2024, using various combinations of the following keywords: “light pollution”, “artificial light at night”, “mental health”, “affective disord*”, “mood disord*”, “anxiety disord*”, “circadian rhythm*”, “sleep disturb*”, “sleep disord*”. We included original research on human samples evaluating the relationship between light pollution and mental health, particularly studies reporting results on affective disorders and affective symptoms, being considered as a possible effect of exposure to artificial light. Studies assessing the effects of light pollution on affective symptoms/affective disorders as secondary outcomes were considered as well. As we hypothesize that circadian disruption is the main mediator of this association, we also included studies that examined the association between light pollution and circadian rhythms. We also included research evaluating risk perception concerning exposure to light pollution to investigate whether this might be another mediator of the emergence of affective symptoms. We excluded reviews, perspective manuscripts, commentaries, letters to the editor, and case reports. Articles published in languages other than English were also excluded. Studies that focused on biological data without clinical measures and studies on animal samples were excluded, as were studies on plants and other organisms.

## 3. Results

### 3.1. Literature Search Results and Study Description

After duplicate removal, literature screening, and hand screening of relevant references, 18 papers were included in the present narrative review. The selected studies variably investigate the role of indoor and outdoor light at night. Data were mainly collected in countries with higher levels of environmental pollution. The studies were grouped according to three main areas of evidence emerging from the literature research: sleep (nine studies), mood disorders and related behaviors (six studies), and risk perception regarding light pollution (three studies). The latter was considered because a survey of people's attitudes towards this issue is fundamental for the development of appropriate prevention strategies in the field of environmental health, as it may also mediate the emergence of new forms of psychopathology [21]. The data extraction of the included papers is reported in Table 1.

### 3.2. Light Pollution, Sleep, and Circadian Disruption

Sleep quality is a crucial determinant of wellbeing, linked not only to individual factors but also to the environment in which the person lives. For this reason, the role of light pollution as a possible moderator of the occurrence of sleep disturbances as an effect of urbanization processes may be crucial. There are currently few studies quantifying the effects of light pollution on overall sleep quality. Research attempted to measure the effect of artificial light on sleep quality by studying both indoor sources of artificial light and outdoor light pollution, e.g., by comparing subjects living in rural areas with those living in urban areas or exposed to different levels of ALAN. To measure the quality of sleep, most studies that were included in this review used the Pittsburgh Sleep Quality Index (PSQI) test [32], but subjective measures, e.g., surveys, were also used.

The possible association between artificial light and poor quality of sleep was confirmed by eight studies [33,34,35,36,37,38,39,40]. One study found small effect sizes, despite evidence of a negative correlation between night-time light exposure and sleep outcomes, suggesting that the effects of ALAN on sleep are more likely to be idiosyncratic [41]. As for outdoor ALAN, a comparative cross-sectional study [37] of adults living in rural and urban areas, where nighttime radiance measurement was considered as a measure of light pollution, found a poorer sleep quality in the urban population compared with the rural population exposed to the same levels of light pollution. Two of the above-mentioned studies [33,36] specifically investigated the effects of indoor night light on sleep. Indeed, artificial lighting is common in bedrooms and individuals often fall asleep with lights on—deliberately or unintentionally—or with the television light on. Similarly, children who are afraid of the dark may ask their parents to keep their lights on during sleep. In particular, one of the included studies investigated the effect of light on sleep quality and brain activity by performing two whole-night polysomnography (PSG) sessions, one with lights off and one with lights on [36]. Lights-on sleep was associated with increased stage 1 sleep, decreased slow-wave sleep, and increased arousal index. Spectral analysis revealed that theta power during REM sleep and slow oscillation, delta, and spindle power during NREM sleep were decreased in lights-on sleep conditions.

Vulnerable age groups were considered as well. In particular, a Chinese population-based cross-sectional study [34] explored the associations between ALAN exposure and sleep disturbances in children; sleep disturbances were measured by the Chinese version of the Sleep Disturbance Scale for Children (SDSC) [42]. Findings from this study suggested that sleep disturbances were more common among children living in areas with high levels of ALAN coming from outdoor sources, and that this exposure is associated with increased odds of sleep disturbances, shorter sleep duration, and longer sleep latency. The associations were stronger in children younger than 12 years, possibly because early childhood is a period when children are more sensitive to light-induced melatonin inhibition, and the sensitivity decreases with age [34]. Area-level outdoor ALAN, as derived from satellite imagery data, was associated with less favorable sleep patterns, mood, and anxiety disorders in adolescents, as found in a cross-sectional study conducted in the US [38]. In this study, area-level outdoor ALAN was also associated with mood disorders for both bipolar and major depressive disorder/dysthymia, while associations with anxiety disorders were driven by specific phobias. In a population of students aged 20–22 years old, an intervention aimed at reducing exposure to night-time artificial light, with a particular focus on blue light emitted by smartphones, led to a significant improvement in sleep quality with a large effect size [39]. In older subjects (age ≥ 60), increasing exposure to ALAN seemed to be associated with increased prescription of hypnotic drugs and daily dose intake (zolpidem and triazolam), as demonstrated by a study conducted in South Korea [35]. Exposure to pre-awake light was also strongly associated with sleep disturbance in elderly subjects in a Japanese cross-sectional study [33]. Long-term exposure to outdoor ALAN was confirmed to increase the risk of poor sleep quality in Chinese veterans in a large study using mixed-effect logistic regression models [40]. The odds of reporting poor sleep quality were higher among participants who suffered from depression.

### 3.3. Light Pollution and Mood Disorders: Depressive, Hypomanic/Manic Symptoms, and Suicidal Behaviors

It has largely been demonstrated that individuals who experience seasonal changes in the length of the day, suffer from jet lag, or work regularly at night are more likely to develop depressive symptoms and mood changes, but it is not known whether outdoor ALAN affects the incidence of depressive disorders, bipolar disorders (BD), under-threshold mood symptoms, or suicide risk in the general population.

#### 3.3.1. Light Pollution, Depressive Symptoms, and Suicidal Behaviors

The studies included in this review suggest that outdoor ALAN is associated with an increased prevalence of depressive symptoms and suicidal behaviors. In two studies [35,43], depressive symptoms were measured using self-administered scales, specifically the Center for Epidemiologic Studies Depression Scale (CES-D) [44] and the Patient Health Questionnaire(9 items) (PHQ-9) [45], while suicidal behavior was defined using an operational criterion as the experience of suicidal ideation or attempt. ALAN exposure assessments were based on earth-observing satellites. Using a large population sample, a study conducted in South Korea found that outdoor ALAN was significantly associated with depressive symptoms and suicidal behaviors [46]. In particular, adults living in areas exposed to higher outdoor ALAN levels showed increased odds of depressive symptoms and suicidal behaviors compared with those living in areas exposed to lower levels. In a study from the Netherlands [43], a statistically significant rise in depressive symptoms was observed with increasing levels of outdoor ALAN in the immediate residential environment, according to an unadjusted model. After adjusting for individual-level and environmental correlates, the association remained significant. A Japanese cross-sectional analysis also provided data on bedroom artificial levels during nighttime as a marker of indoor light pollution, revealing that higher levels were significantly associated with depressive symptoms, measured by the short version of the Geriatric Depression Scale (GDS-15) [33,47]. To explain neurobiological mechanisms through which ALAN may contribute to mood disorders, the disruption of circadian rhythmicity was demonstrated to be critical for mood disorders, including depression, as blue wavelengths at night can also suppress melatonin levels and their production [48]. A large Biobank-based study explored the association between night-time light emission and depressive symptoms, together with its association with urban features, e.g., air pollution, low green space, and poverty [49]. Higher light emission during night-time was associated with higher severity of depressive symptoms, together with higher levels of air pollution, economic and neighborhood deprivation, and higher household poverty. A prospective study evaluated the effects of different sources of artificial light, both indoors and outdoors, on sleep quality in pregnant women referred to nine maternity clinics in Malaysia [50]. Although it was not possible to draw causality from the results of this study, sleep quality and light exposure were shown to affect psychological well-being, with a specific association with anxiety and depressive symptoms.

#### 3.3.2. Light Pollution and Bipolar Disorders

Sleep–wake cycle disruptions and artificial light pollution may also trigger affective episodes in BD. Indeed, BD is associated with the alteration of several circadian rhythms including sleep–wake cycles, energy levels, and physical and social activities [51]. Accordingly, levels of light exposure during early life have been shown to influence later clinical features, such as the age at onset of BD [52]. Lighting conditions may influence the development of affective episodes or exacerbations of clinical symptoms. Particularly, the role of sleep deprivation in inducing mania has already been established. It has also been hypothesized that the blockade of nocturnal production of melatonin caused by ALAN may play a role in the pathogenesis of BD, as a consequence of melatonin’s balancing effect on steroid hormones [53]. There are two studies on indoor ALAN exposure in subjects with BD [54,55], which highlighted the possible contribution of light pollution to the exacerbation of manic symptoms and the recurrence of manic/hypomanic episodes.

### 3.4. Risk Perception Related to the Effects of Light Pollution on Health

#### 3.4.1. Risk Perception of Light Pollution, Sleep Disturbances, and Affective Symptoms

In an Irish study, risk perception of the effects of light pollution among residents was significantly influenced by location, underlining the importance of assessing experiences and attitudes in a wide variety of geographical environments [56]. Indeed, participants in this study confirmed that light entering the bedroom had a detrimental impact on sleep. Moreover, they suggested that light pollution may be a relevant factor to consider when implementing environmental strategies for improving overall sleep health [56]. A cross-sectional study, also conducted in an Irish non-clinical sample, examined how participants perceived ALAN exposure in their sleeping environment and how subjective perceptions of light pollution, as well as objectively measured household illumination levels, might be associated with measures of psychological distress, investigated through the General Health Questionnaire (GHQ) [57]; cognitive failures, studied using the Cognitive Failures Questionnaire (CFQ) [58]; and sleep duration quality and chronotype, measured with the PSQI and the Munich chronotype Questionnaire (MCTQ) [50]. Among respondents, those who perceived outdoor ALAN reported poorer sleep quality, more severe cognitive impairment; higher GHQ scores, particularly for somatic symptoms; anxiety/insomnia symptoms; and depression, when compared to those who did not perceive external ALAN entering the sleep environment [23,59]. To note, authors underlined a mismatch between the levels of ALAN and the subjective perception of ALAN presence in the bedroom during sleep, which meant that the perceived disruptive effect of ALAN on sleep appeared to be, to some extent, altered. Concerning these findings, it should be highlighted that poor sleepers and those with greater psychological distress could be hyper-aware of their sleep quality and thus show a sleep-related attentional bias to specific aspects of their sleep environment. Also, poor sleepers have a higher sensitivity towards light and are subsequently more vulnerable to sleep alterations. Finally, in this study, the perception of light entering the bedroom from indoor sources was not associated with differences in GHQ scores and showed smaller associations with CFQ and PSQI scores than external ALAN did.

**Table 1:** Overview of studies included in the narrative review.

Circadian Rhythms Disruption							
Reference	Country	Design	Sample	Mental Health Outcome	Assessment Measure	Light Exposure (Measure Unit)	Main Findings
Cho et al. [36]	Korea	Cross-sectional study	10 healthy young adults (21–34 years old)	Sleep quality and brain activity during sleep	PSQI PSG	Indoor LAN: fluorescent lamp (40 lux, 30 cm long), approximately 1 m away from participants’ eyes	Increased stage 1 sleep, decreased slow-wave sleep, and increased arousal index during lights-on sleep. Theta power (4–8 Hz) during REM sleep, and slow oscillation (0.5–1 Hz), delta (1–4 Hz), and spindle (10–16 Hz) power during NREM sleep decreased in lights-on sleep.
Lahiri et al. [37]	India	Comparative cross-sectional study	263 participants from urban and 249 participants from rural areas (18–60 years old)	Sleep quality	PSQI 10-item PSS	Outdoor ALAN: nighttime radiance (1 radiance unit = 10^−9^ W/cm^2^/sr)	Poorer sleep quality with higher nighttime radiance exposure. For urban participants, adjusted coefficient of 12.84 (95% CI: 12.31, 13.37) for exposure of >40.0 nW/cm^2^/sr.
Min & Min [35]	South Korea	Population-based cohort study	52,027 older adults from the NHIS-NSC cohort (≥60 years old)	Insomnia	Hypnotic drugs prescription (zolpidem and triazolam)	Outdoor ALAN: satellite mapping of artificial light; light pollution levels (nanowatts/cm^2^/sr)	Regression coefficients for prescription days and daily defined doses of hypnotic drugs were significantly higher among people living in areas with higher outdoor artificial nighttime light.
Obayashi et al. [33]	Japan	Cross-sectional study	2947 adults (≥40 years old)	Sleep quality (PSQI) Depressive symptoms	PSQI GDS-15	Bedroom LAN (median intensity 1.0 lux)	Higher risk for sleep disturbances and depressive symptoms in groups with median LAN intensities ≥ 3 and ≥10 lux (sleep disturbances: OR 1.25, 95% CI 1.05–1.48, *p* = 0.011 for 3 lux; OR 1.29 95% CI 1.02–1.64; *p* = 0.034 for 10 lux; depressive symptoms: OR 1.30, 95% CI 1.05–1.61; *p* = 0.017 for 3 lux; OR 1.33, 95% CI 1.003–1.77; *p* = 0.047 for 10 lux).
Paksarian et al. [38]	USA	Population-based, cross-sectional study	10,123 adolescents; 6483 for behavior disorder outcomes (13–18 years old)	Sleep patterns. Past-year mood, anxiety, behavior, and substance use disorders	Modified version of the CIDI (v. 3.0 according to DSM-IV criteria). Self-reported habitual sleep patterns. Parent-reported information included in behavior disorder diagnoses	Outdoor ALAN, transformed into units of radiance (nW/cm^2^/sr)	Higher ALAN levels associated with later weeknight bedtime. Adolescents in the highest ALAN quartile went to bed 29 (95% CI, 15–43) minutes later and reported 11 (95% CI, 19–2) fewer minutes of sleep than those in the lowest quartile. Positive association between ALAN and prevalence of past-year mood and anxiety disorders: each median absolute deviation increase in ALAN associated with 1.07 (95% CI, 1.00–1.14) times the odds of mood disorders and 1.10 (95% CI, 1.05–1.16) times the odds of anxiety disorders. Association with BD (OR 1.19 [95% CI, 1.05–1.35]) at further analyses.
Patel [41]	USA	Cross-sectional study	282,403 MMSA inhabitants. County level: 2823 inhabitants (≥18 years old)	Low/insufficient sleep	MMSA: self-reports of sleep hours and insufficient sleep. County level: prevalence of insufficient sleep	Outdoor ALAN (nW/cm^2^/sr)	MMSA level: 10-unit increase in nighttime light associated with 5.59 min per day estimated decline in sleep and increase of 13.77% of the odds of reporting insufficient sleep (<7 h). County level: 10-unit increase in nighttime light associated with increase of 2.19% of the prevalence of insufficient sleep.
Randjelovic [39]	Serbia	Interventional study	30 young adults (university students) (20–22 years old)	Sleep quality	PSQI	Blue light emission from LED blacklight screens	Reduction of total PSQI score from 6.83 ± 2.73 to 3.93 ± 1.68 after the intervention (*p* < 0.0001; d = 1.02).
Sun [40]	China	Cross-sectional study	7258 veterans (≥60 years old)	Sleep quality	PSQI	3-year outdoor ALAN exposure (nW/cm^2^/sr)	ALAN exposure above the threshold associated with poorer sleep quality, with OR 1.15 (CI 95% 0.97–1.36) and 1.45 (CI 95% 1.17–1.78) at the 75th and 95th percentiles of ALAN against the threshold. Association of ALAN exposure with poor sleep quality more pronounced in veterans with depression.
Wang et al. [34]	China	Population-based cross-sectional study	20,994 children and adolescents (2–18 years old)	Sleep disorders	SDSC (Chinese version)	Outdoor ALAN exposure from 0.02 to 113.48 nW/cm^2^/sr within 500 m of each participant’s residential address	Higher quintiles of outdoor ALAN exposure associated with an increase in sleep disturbances (total sleep scores) of 0.81 (95% CI 0.66–0.96) in Q2, 0.83 (95% CI 0.68–0.97) in Q3, 0.62 (95% CI 0.46–0.77) in Q4, and 0.53 (95% CI, 0.36–0.70) in Q5. Higher quintiles of exposure associated with OR for sleep disorder of 1.34 (95% CI 1.23–1.45) in Q2, 1.43 (95% CI 1.32–1.55) in Q3 1.31 (95% CI, 1.21–1.43) in Q4, and 1.25 (95% CI, 1.14–1.38) in Q5.
Mood symptoms							
Esaki et al. [55]	Japan	Cross-sectional study	184 subjects with BD (18–75 years)	Manic symptoms in BD patients	YMRS	Indoor ALAN bedroom light exposure (from bedtime to rising time assessed for 7 consecutive days using a portable photometer)	Prevalence of hypomanic states significantly higher in participants with an average light intensity at night exposure of ≥3 lux (36.7% versus 21.9%; *p* = 0.02), with significantly higher OR (2.15, 95% CI 1.09–4.22, *p* = 0.02) at the multivariable logistic regression analysis adjusted for BD type, depressive symptoms, sleep duration, and daytime physical activity.
Esaki et al. [54]	Japan	Longitudinal study	172 subjects with BD (18–75 years)	Mood episode relapses in BD patients	Manic or hypomanic episodes (with or without mixed features) or depressive episodes according to the DSM-5 criteria	Indoor ALAN bedroom light exposure (from bedtime to rising time assessed for 7 consecutive days using a portable photometer)	Risk for manic/hypomanic relapses significantly higher with an average nighttime illuminance ≥ 3 lux (HR 2.54, 95% CI 1.33–4.84); significant relationship even at the multivariable model adjusted for a propensity score in relation to nighttime light (HR 2.17, 95% CI 1.04–4.52). No significant association between nighttime light and depressive relapses.
Helbich et al. [43]	The Netherlands	Cross-sectional survey	10,482 adults (18–65 years)	Depressive symptoms	PHQ-9	Outdoor ALAN satellite-based measures of radiances for exposure assessments (nW/cm^2^/s)	Significantly higher PHQ-9 scores among people in the second to fifth ALAN quintile (β_Q2_ = 0.503, 95% CI 0.207–0.798, β_Q3_ = 0.587 95% CI 0.291–0.884, β_Q4_ = 0.921, 95% CI: 0.623–1.218, β_Q5_ = 1.322, 95% CI 1.023–1.620). ALAN risk estimates adjusted for individual and area-level confounders still significant for the 100 m buffer.
Liao [49]	United Kingdom	Cohort study (secondary analysis of baseline data)	200,393 adults	Depressive and anxiety symptoms; sleep patterns	Diagnostic category in the UKBB. Self-reports for sleep patterns	Five-year mean NLE value	Higher NLE associated with higher depressed mood, higher tiredness/lethargy, and obesity. As for other determinants of mental health, association between higher NLE and higher air pollution, less green space, higher economic and neighborhood deprivation, and higher household poverty. Economic deprivation, household poverty, and waist circumference acting as bridge factors between key urban features and mental health symptoms.
Min & Min [46]	Korea	Population-based, cross-sectional study	113–119 participants for the assessment of depressive symptoms and 152–159 participants for the assessment of suicidal behavior; data from KCHS (≥20 years old)	Depressive symptoms and suicidal behavior	CES-D (Korean version). Questions about attempted suicide or contemplated dying over the preceding 12 months	Outdoor ALAN satellite data from the National Center for Environmental Information (from 0 to 63 nW/cm^2^/sr. Spatial resolution: 50–100 m and detection limit 10^−9^ W/cm^2^/sr)	Higher likelihood of depressive symptoms and suicidal behaviors in participants living in areas with highest ALAN levels (OR 1.29, 95% CI: 1.15–1.46 and OR = 1.27, 95% CI: 1.16–1.39, respectively).
Ng [50]	Malaysia	Prospective study	169 pregnant women (20–48 years old)	Depressive symptoms, anxiety, and stress. Sleep quality	DASS-21 PSQI (second and third trimesters)	Light exposure (H-LEA)	Higher lux level exposed from 10 pm to < 1 am associated with increased stress (β = 0.212, *p* = 0.037) and depression (β = 0.228, *p* = 0.024) in the third trimester. Poor sleep quality and higher light exposure at night attributed to greater stress and depression symptoms in the third trimester. Adverse effect of poor sleep quality on anxiety (β = 0.243, *p* = 0.002) and depression levels (β = 0.259, *p* = 0.001) in the second trimester.
Risk perception							
Cleary-Gaffney et al. [23]	Ireland	Cross-sectional study	552 adults (≥18 years old)	Citizens’ perceptions of ALAN exposure and its impact on psychological distress, cognitive failures, sleep quality, and chronotype	Light at night questionnaire. GHQ, CFQ, PSQI, MCTQ, MSI	ALAN exposure in the sleeping environment	Perception of external ALAN in the sleeping environment associated with poorer sleep quality, cognitive impairment, and greater psychological distress. Internal lighting passing into the sleeping environment associated with poorer sleep quality but not with psychological wellbeing. Habitual use of light-emitting devices associated with poorer psychological wellbeing but not with sleep quality and timing. No associations between the perception of external ALAN and MSI scores.
Coogan et al. [56]	Ireland	Cross-sectional survey	462 adults (age ≥ 18 years old)	Citizens’ perceptions of light pollution and its impact	12-item questionnaire on light pollution	Outdoor ALAN perceptions of recent increase in light at night, the impact of light at night on sleep, changes in the timing of bird song, changes in the night time behavior of animals and changes in the number of bats seen	Perception of brighter night skies in urban settings, with public lighting reported as the main source of light at night. Neighbors’ domestic lighting reported as the most common source of ALAN for rural settings. Respondents from rural settings more likely to report sleep impairment due to ALAN.
Kim et al. [60]	Korea	Cross-sectional survey	1096 research subjects (20–50 years old)	Citizens’ perceptions of light pollution and its impact and social amplification of risk	Survey on environmental and health risk factors, considering psychometric variables that influence risk perception	Outdoor ALAN (light trespass, over-illumination, glare, and light clutter)	Among the 11 environmental risk factors examined in the study, highest rank for light pollution variables reported for glare (5.78 points, fifth position), while over-illumination (5.17 points), light trespass (5.11 points), and light clutter (4.80 points) ranked ninth, tenth, and eleventh. Influence of all psychometric variables except for controllability on risk perception.

Notes: ALAN = Artificial light at night: BD = Bipolar Disorder; CES-D = Center for Epidemiologic Studies Depression Scale; CFQ = Cognitive Failures Questionnaire; CI = Confidence interval; CIDI = Composite International Diagnostic Interview; DASS-21 = Depression, Anxiety, and Stress Scale; GDS-15 = short version of the Geriatric Depression Scale; GHQ = General Health Questionnaire; H-LEA = Harvard Light Exposure Assessment; HR = Hazard ratio; KCHS = Korean Community Health Survey; LAN = Light at night; MCTQ = Munich Chronotype Questionnaire; MMSA = metropolitan and micropolitan statistical area; MSI = Melatonin-suppression index; NHIS = The National Health Insurance Service; NHIS-NSC = NHIS-National Sample Cohort; NLE = Night-time light emission; OR = Odds ratio; PHQ-9 = Patient Health Questionnaire (9 items); PSG = polysomnography; PSQI = Pittsburgh Sleep Quality Index; PSS = Perceived Stress Scale; SDSC = Sleep Disturbance Scale for Children; UKBB = United Kingdom BioBank; YMRS = Young Mania Rating Scale.

#### 3.4.2. Factors Influencing Risk Perception of Light Pollution

A study conducted in Korea [60] used a survey where participants were asked to rate the risk perception level of the selected types of light pollution (light trespass, over-illumination, glare, and light clutter) together with other environmental factors that could represent a major threat to human health. The following factors were selected as indices possibly influencing risk perception: personal knowledge, risk awareness, controllability, impact of the risk on future generations, familiarity with the risk, fatal consequences of the risk, attribution of responsibility, and trust in government. The variables that demonstrated a possible explanatory role were the information obtained from the media and the recall of media criticism. Risk perception of each light pollution type and other environmental risk factors were compared. Among the considered environmental risk factors, glares were significantly more represented than lighting, light trespass, and light pollution. The results also showed that risk perception was considerably influenced by the acquisition of information from the media [60], confirming that media attention towards urbanization-related issues may affect people’s worries about environmental threats and their behavior to reduce them. The recall of media criticism of each type of light pollution led to a statistically significant increase in risk perception [61].

## 4. Discussion

The results of this narrative review point towards an association between light pollution and mental health issues. In particular, increased exposure to light pollution—both indoors and outdoors—appears to have a negative effect on the quality of sleep in populations of different ages, including vulnerable populations, such as adolescents and elderly people. As hypothesized, increased exposure to indoor and outdoor ALAN appears to be associated with the emergence of mood symptoms and with the exacerbation of pre-existing disorders, as demonstrated for BD. The relationship between artificial light exposure and mood disturbances is likely to be mediated by specific biological pathways. In particular, crucial consequences of light pollution that are potentially involved in mood regulation appear to be the decrease in melatonin secretion and disturbances of circadian rhythms [62,63]. Indeed, these latter features play a crucial role in the pathophysiology of mood disorders, both depressive and bipolar [64,65,66,67], and also represent clinical symptoms that significantly impact the phenomenology of these conditions during acute episodes and inter-episodic phases [66,68,69,70,71]. Subsequently, inappropriate or persistent ALAN exposure may desynchronize biological rhythms and exacerbate mood disorders. Furthermore, ALAN has been demonstrated to increase neuroinflammation (for review, see Ref. [72]), a mechanism that has been strongly implicated in the development of psychiatric disorders, particularly mood disorders [73,74]. It is important to note that neuroinflammatory mechanisms are also believed to contribute to the effects of other pollutants, e.g., air pollutants, on the central nervous system, possibly driving the worsening of psychiatric symptoms after short-term exposure [75,76].

Based on the above, novel treatment strategies were also proposed based on the significant role of circadian alterations in the development of mood disorders. In particular, interpersonal and social rhythm therapy (IPSRT) is a psychological strategy that targets biological rhythm disruptions in people with mood disorders [77,78,79]. Interestingly, specific IPSRT protocols targeting vulnerable populations, particularly adolescents, even in at-risk stages, were also developed [78,80,81]. Further non-pharmacological approaches, e.g., psychoeducational approaches, also cover the area of circadian rhythms, discussing with patients the possibility of resynchronizing circadian cycles as a crucial point in the treatment of mood symptoms [82,83,84]. This may be of crucial importance due to the susceptibility of this age group to the effects of environmental factors disrupting biological rhythms, particularly ALAN, which can be emitted by a wide range of electronic devices, such as smartphones [39]. Since integrated approaches are of utmost importance in the field of early intervention, youths suffering from mood symptoms, or even during at-risk stages, should always be proposed to undergo such treatments [85]. Research on vulnerable populations should not be limited to youths, since data from this review also show specific susceptibility in older adults. This is of particular interest since poor sleep quality has been demonstrated to significantly worsen the quality of life in elderly populations, also mediating the onset of mood disorders, particularly depression [40,86]. Subsequently, in this population, the exposure to sources of light pollution should be systematically evaluated as well, and psychoeducational interventions should be further developed, together with environmental strategies aimed at risk reduction.

This narrative review also evidenced that risk perception concerning ALAN could influence the emergence of psychological distress in response to this specific exposure. This is a controversial issue since it has been largely argued that the perception of risk related to environmental stressors may even lead to the development of new forms of psychopathology and should thus be evaluated in all exposed subjects [21,61]. To note, eco-anxiety might to some extent also have moderated the effects of light pollution on the development of affective symptoms. Indeed, this novel psychological feature was shown to be associated with the development of depression, especially in vulnerable populations [87]. Further research is expected to clarify the possible impact of light pollution in the development of eco-anxiety, as well as the relationship of this feature with mood symptoms in people exposed to ALAN. On the other hand, low-risk perception of light pollution, which could be due to insufficient interest in environmental determinants of health and to underestimation of artificial light consequences on human health, may lead to a number of unhealthy habits [88]. As it is predicted that light pollution could pose a growing, serious potential risk in the future [89], it is necessary to develop strategies to inform the public about the risks of light pollution. Therefore, an effective communication strategy should be implemented, e.g., by improving the dissemination of information through the Internet and social media.

The main strength of the studies here included, especially those on mood disorders, is the representative number of participants enriched with individual-level register and environmental data. At the same time, some limitations should be underlined. First, the analyses were mainly based on self-reported depressive symptoms, which represents a multi-faceted issue. Indeed, patient-reported outcomes are a main goal in the field of mental health and self-administered scales allow for reporting symptoms that may not be objectively observed by clinicians. On the other hand, these measures may have introduced some biases into the analyses because any symptoms would deserve to be further discussed with clinicians. It should also be pointed out that different sources of pollutants contribute to the complexity of the urban environment, which is a major threat to human mental health and may also include a number of social stressors [90]. To note, light pollution and artificial lighting have also been advocated as having social effects that should be considered an integral part of this complexity [89]. Another limitation of the included studies is that individual sleeping habits were not considered in most cases and altered sleep hygiene was not specifically assessed. This could represent a further bias, as it is not always possible to distinguish between changes in people's sleep habits and the effects of light pollution. Several studies included adults of different ages, which could also have introduced some bias since sleep habits sensitively change during the lifespan. Similarly, research was conducted at different latitudes where times of sunrise and sunset highly vary, as well as light quality and intensity. This could have significantly affected the duration of ALAN exposure and limited the generalizability of findings. Finally, most of the included literature focused on population samples rather than clinical ones. The latter would require further interest in the near future, also in consideration of the results of this review. On the basis of the revised literature, it is crucial to perform studies with robust designs and with particular interest in longitudinal data collection. As for vulnerable populations, there is a need for interventional studies specifically aimed at understanding what preventive strategies can be put in place to avoid the onset of clear-cut psychopathology in the event of exposure to environmental stressors. Finally, a wide range of urbanicity-related factors should always be considered when assessing human health, particularly mental health, in the urban environment. Together with this, identifying social variables and attitudes that affect the risk perception of any type of pollution represents a priority for the implementation of appropriate prevention strategies.

## 5. Conclusions

In this present narrative review, we summarized the possible detrimental effects of light pollution on mental health, with evidence of the role of artificial light at night in the development of circadian disruptions and mood symptoms, together with the exacerbation of mood disorders. Light pollution should be considered among the determinants of mental health in the context of the urban environment. Research into mood disorders in the post-modern era should not underestimate the effects of urbanization and should thus be aware of the detrimental effects of various sources of pollutants on the development and exacerbation of these conditions. This relationship is complex and should take into account factors such as individual exposure and risk perception. Although future studies are expected to further clarify and characterize the effects of light pollution on mood disorders and the moderators of this association, the results of this review suggest how building more livable cities that protect public health should include eco-friendly lighting, alongside other factors, according to the principles of sustainability. Furthermore, raising people’s awareness of risk factors and harmful habits related to light-dependent circadian rhythms should be a public health priority.
